# A major QTL affects temperature sensitive adult lethality and inbreeding depression in life span in *Drosophila melanogaster*

**DOI:** 10.1186/1471-2148-8-297

**Published:** 2008-10-28

**Authors:** Cornelis J Vermeulen, R Bijlsma, Volker Loeschcke

**Affiliations:** 1 Ecology and Genetics, Department of Biological Sciences, University of Aarhus, 8000 Aarhus C, Denmark; 2 Population & Conservation Genetics, Evolutionary Genetics, University of Groningen, P.O. Box 14, 9750 AA Haren, Netherlands

## Abstract

**Background:**

The study of inbreeding depression has major relevance for many disciplines, including conservation genetics and evolutionary biology. Still, the molecular genetic basis of this phenomenon remains poorly characterised, as knowledge on the mechanistic causes of inbreeding depression and the molecular properties of genes that give rise to or modulate its deleterious effects is lacking. These questions warrant the detailed study of genetic loci giving rise to inbreeding depression. However, the complex and polygenic nature of general inbreeding depression makes this a daunting task. Study of inbreeding effects in specific traits, such as age-specific mortality and life span, provide a good starting point, as a limited set of genes is expected to be involved.

**Results:**

Here we report on a QTL mapping study on inbreeding related and temperature sensitive lethality in male *Drosophila melanogaster*. The inbreeding effect was expressed at moderately high temperature, and manifested itself as severe premature mortality in males, but not in females. We used a North Carolina crossing design 3 to estimate average dominance ratio and heritability. We found the genetic basis of the lethal effect to be relatively simple, being due mainly to a single recessive QTL on the left arm of chromosome 2. This locus colocalised with a QTL that conditioned variation in female life span, acting as an overdominant locus for this trait. Male life span was additionally affected by variation at the X-chromosome.

**Conclusion:**

This demonstrates that analysis of large conditional lethal effects is a viable strategy for delineating genes which are sensitive to inbreeding depression.

## Background

Inbreeding depression is defined as the decrease in fitness related characters in offspring resulting from matings between related individuals. This phenomenon is known to have large impact on fitness, adaptive ability and extinction probability of populations, and has an important role as a selective force in the evolution of mating systems and dispersal strategies [1-3]. Furthermore, it is of major relevance in animal and plant breeding, conservation and evolutionary biology [4,5]. It is believed that inbreeding depression is due to the expression of multiple deleterious recessive alleles, as a result of increased homozygosity. If the expression of some deleterious alleles is conditional on specific environmental conditions, this can result in an abnormally high sensitivity for environmental challenges, in addition to a general decrease in fitness [6,7].

Although inbreeding depression is well studied at the phenotypic and population level, knowledge on the mechanistic genetic basis of inbreeding depression is still very scant. Only recently have powerful tools become available to detect loci that underlie inbreeding depression [8] and to characterise the changes that occur at the molecular and biochemical level during inbreeding depression [9-11]. For example, it is unknown whether inbreeding effects in certain traits will consistently map to genes involved in the same pathways across different populations, which is expected if certain genes are more likely to generate recessive deleterious alleles [12]. Also, whether genetic variation among inbred lines is due to the same loci that condition genetic variation in the ancestral outbred population remains an open question. Finally, the deleterious effects of these disruptions are ameliorated by a set of uncharacterised genes that transcriptionally respond to inbreeding [10,11]. These questions warrant characterisation of the genes causing and modulating inbreeding depression.

Inbreeding depression is known to occur in virtually every fitness component, but life span is a particularly interesting character [13]. Life span can be curtailed by the expression of deleterious alleles that may affect age-specific survival during a specific time window [14]. Genes that harbour high frequencies of deleterious alleles may therefore correspond to the QTL affecting genetic variation in life span described in several mapping studies [15,16]. The timing and age-specificity of deleterious alleles are properties that are central to the evolutionary theories of ageing, mutation accumulation and antagonistic pleiotropy [17,18] and are essential for an understanding of the evolution of survival patterns, e.g. late-life mortality plateaus [19].

**Figure 1:**
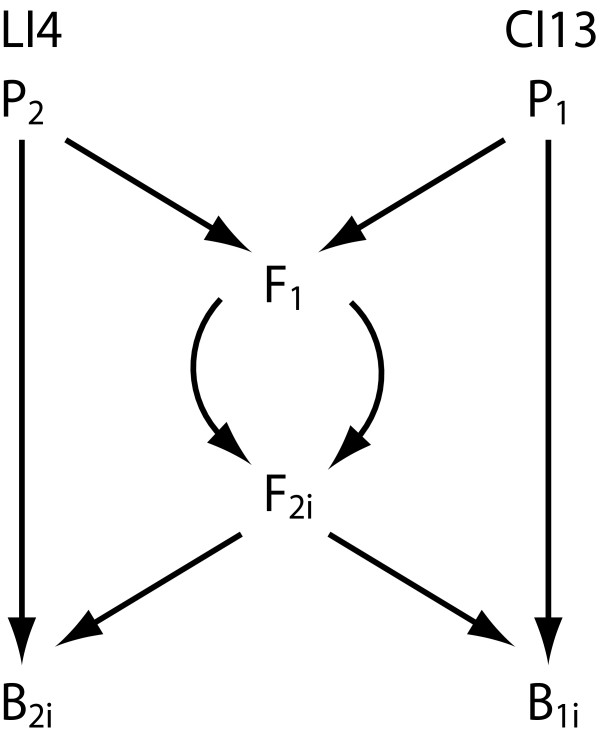
**North Carolina 3 Design employed for mapping QTL**. The P_1 _line indicates the control line (CI13), whereas the P_2 _line indicates the line expressing the lethal effect (LI4). These lines were reciprocally crossed to produce the F_1 _generation, which was crossed among itself to produce the F_2 _generation. Individual F_2_-males (i = 1 to 240) were backcrossed to both P_1 _and P_2 _and thereafter frozen until marker genotype was scored. The resulting backcross progeny (B_1i _and B_2i_) was placed at restrictive conditions to assess longevity and expression of the lethal effect. As reference material, the parental lines and both reciprocal F_1 _crosses were also assessed (not shown).

In this paper we focus on an inbred line of *Drosophila melanogaster *displaying temperature sensitive age-specific adult lethality. Conditional adult lethal effects are encountered regularly in inbred populations of *Drosophila *[20-22]. The study of conditional lethality has several practical and conceptual advantages. First, it focuses on large and reproducible inbreeding effects, which have been characterized in terms of age-specificity, temperature sensitive periods and restrictive conditions [23]. Second, as the lethal effect is very specific, it presumably has a simple genetic basis. The lethal effect studied in this paper has sex-limited expression, affecting only males, and is induced and expressed only in adult flies [23]. As genetic details were lacking, we here aim to complement these data by means of QTL mapping. To establish the mapping population, we used a North Carolina Design 3 (NCD3), in which individual F_2 _males were backcrossed to females from both parental lines (Figure 1). This design allows accurate scoring of the lethal phenotype and is well suited for estimating the dominance ratio. This research was meant to put a lower limit on the number of loci that conditioned the lethal effect, give estimates about gene action (i.e. dominance ratio) and reveal the approximate position(s). The latter may ultimately aid us in identifying the actual gene(s). We also measured female life span, to assess whether the same QTL conditioned life span in females.

## Results

### Phenotypic Description and Variance Analysis

**Figure 2:**
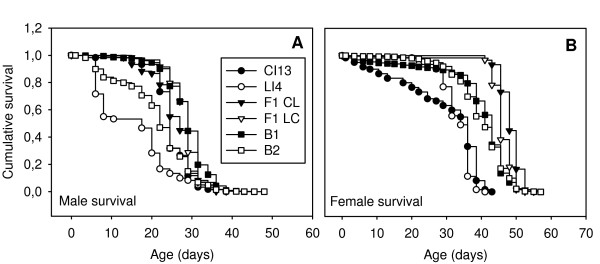
**Survival curves of control and backcross populations**. Curves depict male (A) and female (B) flies. CI13 and LI4 are the pure control and lethal line respectively. LC (lethal female × control male) and CL (control female × lethal male) denote the reciprocal F_1 _crosses. B_1 _and B_2 _denote the mapping populations derived from F_2 _males crossed to control females and lethal females respectively. Curves were established at 29°C with for control populations n = 60 and for backcross populations n > 4700 individuals. Note that the curves for CI13, LC, CL and B_1 _overlap in the first panel.

In the mapping population, mean life span at 29°C was 25.4 ± 0.08 (s.e.) days for males and 40.0 ± 0.09 (s.e.) days for females. The control crosses clearly showed conspicuous expression of the male lethal effect between day 5 and 12, whereby the lethal behaved as a recessive (Figure 2A). This period is called the lethal phase. No excessive mortality was observed in LI4 females during this interval (Figure 2B), confirming previous results [23]. As judged by the mortality pulse during the lethal phase, the lethal effect was also expressed in several families in the backcross to LI4, allowing us to map the effect (see Figure 2A). These crosses allowed estimation of variance components using ANOVA (Table 1). For males, the average dominance ratio was 0.95, signifying almost complete dominance. The heritability in the narrow sense was 0.53, showing that approximately half of the phenotypic variance in the cross was additive genetic. For females the average dominance ratio was 1.93, indicating overdominance (F-ratio test: *F*_238,238 _= 3.1; *P *< 0.001). The heritability in the narrow sense was found to be 0.31. Females from the control line were found to display some premature female mortality (Figure 2B). This clearly shows that inbreeding effects on age-specific survival are pervasive. Since this effect is not the focus of this study and did not further interfere with our analysis, it was ignored.

**Table 1:** Estimates of genetic parameters in the crossing design

Sex	Life span ± s.e.	*V*_*A*_	*V*_*D*_	*V*_*E*_	DR	*h*^2^
Females	40.0 ± 0.09	39.3***	73.2***	12.6	1.93	0.31
Males	25.4 ± 0.08	45.3***	20.5***	19.8	0.95	0.53

Mean life span with standard errors in days, additive genetic variance (*V*_*A*_), dominance variance (*V*_*D*_), environmental variance (*V*_*E*_), average dominance ratio (DR) and narrow sense heritability (*h*^2^). *** significant at *P *= 0.001

### Number of QTL, positions and gene effects

**Figure 3:**
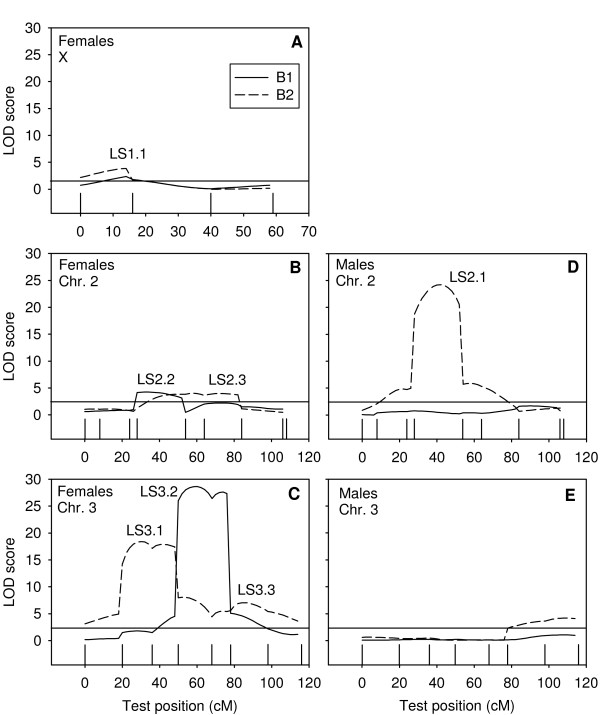
**LOD scores for life span**. Graphs depict LOD scores for the major chromosomes, when testing for QTL affecting female (A, B and C) and male (D and E) life span. For each sex, the two mapping populations are depicted. B_*i *_denotes the mapping populations derived from F_2 _males crossed to control females (i = 1) or lethal females (i = 2). If LOD scores exceed the significance threshold (horizontal line in figure), this indicates a QTL is segregating for life span at that position. QTL names correspond to those used in Table 2. The ticks indicate marker positions.

**Figure 4:**
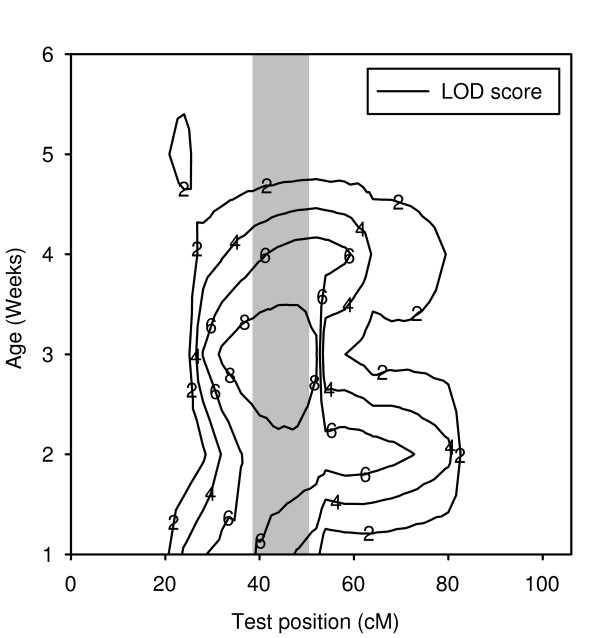
**LOD scores for age-specific male mortality at chromosome 2**. The contour plot shows LOD scores for age-specific mortality in males, as a function of chromosomal position and age (in weeks). The grey bar indicates the 2-LOD interval for the life span QTL designated LS2.1 (the presumed lethal). Significance thresholds vary between weeks from 2.3 to 3.1. Note that the QTL continues to condition age-specific mortality up until and including week 4.

We found multiple QTL conditioning life span and age-specific mortality on all chromosomes. As we are lacking the resolution to perform fine-mapping of all loci, we adopted a conservative approach to detect QTL. We listed all QTL that were detected both by the single marker analysis and the composite interval mapping, and which had peaks separated by at least one marker interval (Table 2). One of our aims was to put a lower limit on the number of loci responsible for the lethal pulse in our line. Because the lethal effect has a profound effect on life span, the responsible loci should show up as QTL for this trait. Male life span in our mapping experiment seemed to be conditioned by a single locus on the left arm of chromosome 2 (Table 2; Figure 3D). We verified that this QTL conditioned the lethal effect, by ascertaining that its position corresponded to that of QTL affecting age-specific mortality in the lethal phase. We found that the locus indeed coincided with QTL affecting weekly age-specific mortality during, and beyond, the lethal phase (Figure 4). The lethal allele was responsible for increased mortality in week 1, 2, 3 and 4 and thus continued to segregate variation well beyond the lethal phase (~between day 5 and 12; Figure 2A). In addition to this major QTL, two minor loci were found to condition premature mortality in week 1, but not thereafter (data not shown).

**Table 2:** Description of the life span QTL

QTL	Peak	2 LOD interval	sex	*a*	*d*	*d*/*a*	V_P _explained
LS1.1	6A-B	1A – 10F	females	0.2	**1.8**	8.39	0.01
LS2.1	31F-32B	30F – 35B	males	**4.8**	**5.1**	1.06	0.21
LS2.2	30E	29C – 36A	females	-0.1	**2.3**	29.84	0.01
LS2.3	49F-50D	45A – 52F	females	-0.9	**3.6**	4.07	0.03
LS3.1	66D	65B – 67C	females	**2.9**	**2.1**	0.73	0.04
LS3.2	85E-86B	73D – 87E	females	**-2.6**	**4.9**	1.89	0.08
LS3.3	94D	92F – 98B	females	0.2	**2.6**	17.03	0.01

QTL name, cytological position with peak and 2 LOD interval, sex affected and estimates of additive (*a*) and dominance (*d*) effects on life span (in days), dominance ratio (*d*/*a*) and proportion of phenotypic variance V_P _explained by the QTL.

Negative values of *a *indicate the LI4 line has the increasing allele at this QTL. This was accounted for in the calculation of the dominance ratio. Significant QTL effects (at *P *< 0.05) are shown in boldface. All QTL shown are supported both by composite interval mapping and single marker analysis.

We tested whether the reciprocal F_1 _crosses were significantly different, both for males and females. Reciprocal crosses of females were not different (*t*_118 _= 1.87, n.s.), dismissing maternal effects as an important factor. However, males from the reciprocal crosses did show a significant difference (*t*_117 _= 2.82, *P *< 0.01), suggesting some X-linked QTL. The X-chromosome of the LI4 line conferred superior longevity (+2.4 days). Our design cannot further delineate these X-linked QTL affecting male life span, as F_2 _sires do not transmit the X-chromosome to their backcross sons.

The estimate of average dominance ratio in female life span suggested one or several overdominant loci. Indeed, we detected a minimum of six major QTL segregating for female life span, and all but one of these behaved as overdominant loci (Table 2). Note however, that our experiment lacks the resolution to exclude the possibility of pseudo-overdominance, which occurs when two partially dominant loci are linked in repulsion phase, inflating estimates of dominance effects. Since many loci seem to condition female life span, this most likely is the case in our experiment.

### Correlation between male and female life span

There was a significant correlation between male and female life span within the B_2 _backcross to the LI4 lethal line (*r *= 0.413, *P *< 0.001), suggesting a shared genetic basis. However, no such correlation was observed within the B_1 _backcross to the control line (*r *= 0.066, n.s.). We wanted to determine whether the lethal allele was responsible for genetic variation in female life span too, even though female flies do not show a lethal phase (Figure 2B). Within the B_2 _backcross there was an overlap between the major male QTL (LS2.1, the presumed lethal allele) and one of the QTL affecting female life span (LS2.2, see Figure 3B). We cannot rule out that this may involve linkage rather than a common genetic basis, a problem that needs to be resolved with further fine mapping. The genetic region that in our study was associated with a clear deleterious effect in males, caused overdominance in females. Within the B_1 _backcross there appeared to be no major QTL segregating for male life span, explaining the lack of correlation between male and female life span.

## Discussion

### A single major QTL underlies the lethal effect

The genetic determination of inbreeding depression is assumed to be highly complex, due to its polygenic nature. This will generally be true for complex traits, like fitness. Inbreeding depression in specific traits however is expected to have lower genetic complexity, and may serve as a simplified model. Because of its conditional nature, we assumed the lethal effect in the LI4 line to be conditioned by a single locus, even though it was already clear from preliminary genetic data that expression was extremely sensitive to genetic background [23,24]. For the LI4 line, this assumption is largely confirmed here. There appears to be a single major locus that conditioned male life span and the lethal effect on the left arm of chromosome 2, with only few minor loci involved when considering age-specific mortality. It is possible that a single gene is causing the lethal effect. However, the QTL region is very large, and we also allow for the possibility that the QTL consists of several linked genes.

### Dominance ratio

Traditionally, two main hypotheses exist to explain inbreeding depression; partial dominance theory and overdominance theory. According to the first theory, inbreeding depression is the result of unidirectional dominance at a large set of loci [25]. A common biological interpretation is an increased expression of rare recessive deleterious alleles during inbreeding. The overdominance theory states that heterozygotes at any locus have superior fitness over either homozygote leading to depressed character values as heterozygotes are lost [26]. The relative contribution of partial *vs*. overdominant loci is still unresolved, although both types of gene action have been found to affect inbreeding depression and heterosis [27-31].

The dominance ratio of the lethal was estimated to be 1.06, which corresponds to the value of 0.95 found by the variance analysis. These data suggest the lethal allele to be a recessive deleterious allele, which fits the partial dominance theory best. However, since the lethal behaved as a partially dominant allele when the I4 line was crossed to an outbred stock, this result may be particular to the genetic background explored in this study [23]. Since only a single large effect QTL was detected, epistasis does not enter into the discussion, although we cannot rule out that significant epistasis is present. We have circumstantial evidence that the genetic background is of importance to the severity of the lethal effect [24] and we intend to study molecular interactions in more detail in subsequent research.

### Genotype by sex interaction

Due to differences in the expression of genes and selection pressures experienced, the build up of inbreeding load may proceed differently between the sexes. Sex-specific differences in inbreeding load have been reported previously, although not often for life span [32]. Our initial analysis suggested that expression of the LI4 lethal effect was limited to male flies [23]. In this study we were able to test whether the QTL responsible for the lethal effect in males also segregated variation for life span in females. We did indeed find a QTL affecting female life span at the same position as the male lethal. QTL mapping often shows many loci to have effects limited to a single sex [33,34], whereas QTL studies in lines artificially selected for increased life span tend to detect QTL that affect both sexes in the same direction [15,35]. The QTL in our study behaved as an overdominant locus in females. This suggests a mechanism whereby this lethal allele may avoid purging in a natural population. If the allele itself has pleiotropic beneficial effects in females, or is tightly linked to a beneficial allele, this may partly counteract the deleterious effects in males, and delay its purging. Whether this can result in a stable polymorphism is unclear, as the interplay between sex-dependence, G × E interaction and gene action complicates this scenario [36]. However, if such effects prove to be common, they may contribute appreciably to the maintenance of the inbreeding load.

### Implications for ageing research

According to the theory of mutation accumulation, ageing is the result of an accumulation of alleles with distinct late-age deleterious effects [17]. Although the mortality effect of the lethal described in this study occurs too early to consider it part of the variation for physiological ageing, the data show that the lethal allele continues to cause excess mortality after the lethal phase. Presumably this reflects mortality of "escaper flies" that have initially survived the lethal phase. Since the lethal allele has no strict age-specific window, weaker alleles may exist that potentially affect life span in outbred populations. In addition, the same QTL conditions age-specific mortality at advanced ages in females. This establishes "inbreeding loci" as candidate genes for genetic variation in life span in outbred populations. Several of the more powerful studies of genetic variation affecting life span and ageing crucially depend on the crossing and analysis of highly inbred lines, e.g. [15,16,34]. It is unknown how inbreeding affects the expression of longevity genes, or whether ageing in inbred lines still is physiological, i.e. not simply curtailed by a disruption of homeostasis. Further investigations are needed to determine whether QTL affecting life span are the same in inbred and outbred populations.

### Candidate genes

The ultimate aim of this study is to identify the main QTL at the molecular level. The position of our lethal does not correspond to that of *l(2)hs*, a heat-sensitive allele previously found segregating in our G83 base stock [21,24]. However, the QTL region contains many candidate genes. A concise list of genes in the region that are implied in life span determination includes *chico*, *DNA methyltransferase 2*, *Sir2*, *tamas*, *Target of rapamycin*, *bubblegum*, *Myosin 31DF*, *porin *and *Pten*, whereas *Hsp60D *is involved in the response to heat. It has been demonstrated that genes with large mutational effects on longevity are not necessarily segregating variation in natural populations [37]. Therefore, other genes in the interval still may correspond to our lethal. We also compared the position of our lethal to life span QTL from several other mapping studies and indeed found some overlap with previously established QTL, e.g. [33,35]. However, given the large confidence intervals in QTL mapping, it is impossible to verify whether these involve the same loci. Confirmation will have to await the identification of the responsible genes.

## Conclusion

It is not our intention to generalise the findings of this single instance of inbreeding depression to the general genetic basis of inbreeding depression. However, by focusing on well characterised effects in a defined trait, instead of general inbreeding depression in fitness, we have shown that it is possible to identify major genetic factors and these will likely be amenable to detailed molecular characterisation. This type of data can enable powerful studies that can provide complementary insights, e.g. whether inbreeding in life span will map to candidate genes known to affect longevity. In addition, since stress responsive mechanisms are known to influence life span [38], it will be interesting to see whether the deleterious effects of inbreeding in life span in our model are buffered by known stress responsive genes. In support of this notion, it has been shown in a genome-wide gene expression study [10,11] that several stress responsive genes were transcriptionally regulated in response to inbreeding. Combined with genomic and quantitative approaches, this will allow a powerful dissection at the cellular, organismal and population level and contribute to a deeper understanding of inbreeding depression.

## Methods

### Stocks

The inbred line I4 was chosen because it displays a high level of early onset adult mortality. Discovery of this lethal effect and the origin of the line are described elsewhere [22]. In brief, inbred lines were established by seven generations of brother-sister mating (F = 0.785). In order to facilitate maintenance after establishment, lines were selected for high productivity during inbreeding. Thereafter, lines have been maintained at large numbers (>300 individuals). The I4 lethal effect was discovered during a study of temperature effects on life span. It is expressed at elevated temperature (maximal at 29°C) and causes severe male mortality (~80%) in the first two weeks of adult life. As a control we chose inbred line I13, which was established at the same time as I4, but which displays high levels of male adult survival both at restrictive and permissive conditions. Both lines were established in 1997. The mortality effect of I4 was first demonstrated in 1999, two years after establishment, indicating that the genetic constitution underlying this effect is stable.

Lines with high levels of homozygosity are desirable in QTL-mapping designs, as this maximizes linkage disequilibrium between QTL and markers. For this purpose, we established highly inbred lines from I4 and I13 by performing 7 additional generations of inbreeding (expected F ~0.95). These lines will be referred to as LI4 (Lethal Inbred) and CI13 (Control Inbred), as to distinguish them from their progenitor stocks.

### Maintenance and experimental rearing conditions

Lines LI4 and CI13 have been maintained in large numbers (> 300 individuals) in quarter-pint bottles (36 ml Leeds medium: 60 g dead yeast, 40 g sugar, 16 g agar, 30 g oatmeal, 16 ml nipagine solution and 1.2 ml acetic acid per litre) at 25°C and 40–60% relative humidity. For assay of adult survival we supplied the food medium with ampicillin (100 mg/L) to avoid bacterial growth.

### Microsatellite analysis

For the contrast LI4 vs. CI13 we used microsatellite markers to track segregation of the parental genomes (See Additional file 1). Primer sequences for microsatellite markers were taken from several web-based resources (Christian Schlötterer laboratory http://i122server.vu-wien.ac.at/Microsatellite%20Loci/Loci%20Titelpage.html, Charles Aquadro laboratory and David Goldstein microsatellite page). The URLs of the latter two websites are no longer available, but information on some of these markers has been published [39-41]. We succeeded in covering the entire genome, using 21 markers. The average spacing between markers was 14 cM, and the estimated material outside the distal markers is 4 cM on average. The lines were fixed for alternative alleles, providing perfect segregation information. The coverage was not dense, but several well-spaced markers suffice to establish the number of QTL and approximate position [42].

Genomic DNA from individual F_2 _males was isolated by the CTAB method [43] and dissolved in 200 μL TE. The PCR were run in 6 μL using 3 pmol of each primer, with the forward primer being Cy5 end-labelled, and one μL DNA. The PCR conditions were 3 minutes at 94°C for initial denaturation, then 40 cycles of 94°C for 30 seconds, annealing temperature (as appropriate for each primer set) for 40 seconds, 72°C for 40 seconds and finally ending with 72°C for 5 minutes. The PCR products typically were diluted 20 times and one μL of diluted product was run on an ALF-express automated sequencer (Amersham Biosciences) together with the internal standard ALF-express™ sizers (100 and 300 bp) and the external ALF-express™ sizers (50 – 500 bp).

### Crossing design

Traditionally, inbreeding depression is analysed by contrasting inbred lines with the ancestral outbred population. We chose not to pursue this strategy, as we aimed to detect the genetic basis of a specific lethal effect, rather than general inbreeding. Instead, we used an inbred control line, which maximizes the power of the mapping design. We decided to employ a half-sib mating test design based on a North Carolina Design 3 (NCD3) [44]. In this design, individual F_2 _males are backcrossed to both parental lines. After mating, the F_2 _males are frozen and genotyped, whereas their backcross progeny provide the phenotypic data (Figure 1). The advantages of this design are an increased accuracy in scoring the phenotype, which has incomplete penetrance, and a powerful design to estimate the dominance ratio. The parental cross was initiated with CI13 (P_1_) and LI4 (P_2_) flies. We set up several single-pair vials at 25°C for each reciprocal cross (P_1 _× P_2 _and P_2 _× P_1_), discarded the parents and allowed F_1 _flies to hatch. Thereafter, ten bottles containing 5 F_1 _females and 5 F_1 _males from each reciprocal cross (totalling 20 individuals) were set up. Simultaneously, culture bottles were set up for each parental line using normal culturing procedure. This set consisted of 2 subsets, offset by two days to allow for transfer of males to "fresh" females in the backcrosses (as described below). To initiate the backcrosses, we collected F_2 _males as well as virgin females from each of the respective parental lines. All were kept separate in yeast-supplied vials for 3 days at 20°C. Thereafter, F_2 _males were individually placed in vials containing either three P_1 _or P_2 _virgin females. In addition, we set up control families with males collected from the parental stocks. We set up equal amounts of B_1_- and B_2_-crosses. After 2 days males were aspirated into the alternative cross-vial with fresh females. The food-tablets with eggs were transferred to 68 ml plastic bottles with 21 ml of standard medium, in order to avoid crowding effects. Two days later, females were discarded and males were removed and stored at -20°C. As in the first set, food tablets were transferred to plastic bottles. Backcross progeny of 240 families was collected.

### Life span assay

For every family, 20 males and 20 females were collected as virgins and stored in single-sex vials containing ten individuals (4 vials per backcross family). These were placed at 29°C, refreshed at day 2 and thereafter twice per week until all flies were dead. Dead flies were scored three times per week. Individual life span was taken as the midpoint of the scoring interval in which the fly died. We lost 0.4% of the experimental flies due to escapes and accidental deaths.

### Statistical analysis

Analysis of variance of the North Carolina Design 3 cross was performed on the life span data as described by Kearsey and Pooni [42] following the original design of Comstock and Robinson [44]. We fitted the model:

(1) *Y*_*ijl *_= *m *+ *T*_*l *_+ *s*_*j *_+ (*Ts*)_*jl *_+ *e*_*ijl *_

where *y*_*ijl *_is the life span of offspring *i *of sire *j *tested against tester *l*, *m *is the intercept, *T*_*l *_is the effect of tester *l*, *s*_*j *_the effect of sire *j*, *(Ts)*_*jl *_the interaction of sire *j *against tester *l *and *e*_*ijl *_is the random error associated with offspring *i *with sire *j *tested against tester *l*. Partitioning of mean squares (MS) allows estimation of additive variance, dominance variance and environmental variance (*V*_*A*_, *V*_*D *_and *V*_*E *_respectively). Assuming no epistasis or gametic phase disequilibrium is present, average dominance ratio (DR) is given by:

(2) DR = √(4 *V*_*D*_/2 *V*_*A*_)

with *V*_*D *_being the dominance variance and *V*_*A *_the additive variance (at equal allele frequencies) [42]. The heritability in the narrow sense (*h*^2^) is given by:

(3) *h*^2 ^= *V*_*A*_·(*V*_*A *_+ *V*_*D *_+ *V*_*E*_)^-1^

with *V*_*E *_being the environmental variance [42]. All statistical analyses were carried out in JMP 6.0.0.

### QTL-mapping procedure

In addition to mean life span, we analysed weekly age-specific mortality (*μ*_*x*_), as estimated by

(4) *μ*_*x *_= -ln(*P*_*x*_)

where *P*_*x *_is the proportion of surviving individuals from week × to week x+1. Mortality *μ*_*x *_was determined separately for each week and calculated on a per family basis. Thereafter, we used *μ*_*x *_as a phenotype for QTL mapping. Data on life span and weekly mortality were analysed in WinQTLCart/QTL Cartographer [45,46], using composite interval mapping. QTL Cartographer did not support the NCD3 testcross design. Because the F_2 _sires were genotyped in our design, the logical solution was to analyse the backcrosses separately assuming an F_2 _design to detect QTL locations. The program was run using default settings (model 6, window size 10 cM, walk speed 2 cM and 5 background markers). The program provides QTL peaks and 2-LOD support intervals, which have a high probability (~95%) of containing the QTL [47]. Significance thresholds at 5 percent were established using Churchill-Doerge permutation tests with 1000 permutations [48]. Map distances in our experiment corresponded to within 6 cM to published values. We performed QTL mapping both with published and calculated marker positions, and both methods yielded similar results. In this paper we present the data based on calculated map distances. We tested all markers for segregation distortion and found a slight excess of heterozygotes at two linked marker loci (SU(VAR); *X*^2 ^_2 _= 6.23, *P *= 0.045 and BIB; *X*^2 ^_2 _= 7.20, *P *= 0.027). We estimated additive (*a*_*k*_) and dominance (*d*_*k*_) effects for QTL *k*, *k *= 1,..., *n *by pooling information from both backcrosses and fitting an additive linear model:

(5) $$y_{il}=m+T_{l}+\sum_{k=1}^{n}(x_{aik}a_{k}+x_{dik}d_{k})+e_{il}$$

where *y*_*il *_is the observation for family *i *in the cross to parental line *l*. Furthermore, *m *is the intercept, *T*_*l *_is the effect of crossing to tester line *l*, *x*_*ai *_and *x*_*di *_are the explanatory variables of *a*_*k *_and *d*_*k *_which depend on flanking marker genotype and backcross type, and *e*_*il *_is the residual term [42,49].

X-linked and carry-over effects will affect these estimates, so we tested whether the reciprocal F_1 _crosses of the control families were significantly different using a t-test.

## Authors' contributions

CJV designed and performed the experiment, analysed the results and wrote the manuscript. RB made, and performed initial characterization of, the I4 and I13 progenitor lines, provided guidance throughout the study and helped writing the manuscript. VL participated in the design of the study, supervised the experiment and helped writing the manuscript. All authors read and approved the final manuscript.

## Supplementary Material

Additional file 1 **Microsatellite list.** Contains microsatellite names, accession numbers, cytological locations, genetic locations, repeat type, references and allelic size ranges. Click here for file
